# BioJS: an open source standard for biological visualisation – its status in 2014

**DOI:** 10.12688/f1000research.3-55.v1

**Published:** 2014-02-13

**Authors:** Manuel Corpas, Rafael Jimenez, Seth J Carbon, Alex García, Leyla Garcia, Tatyana Goldberg, John Gomez, Alexis Kalderimis, Suzanna E Lewis, Ian Mulvany, Aleksandra Pawlik, Francis Rowland, Gustavo Salazar, Fabian Schreiber, Ian Sillitoe, William H Spooner, Anil S. Thanki, José M Villaveces, Guy Yachdav, Henning Hermjakob

**Affiliations:** 1 The Genome Analysis Centre, Norwich Research Park, Norwich, NR4 7UH, UK; 2 European Bioinformatics Institute EMBL-EBI, Hinxton, CB10 1SD, UK; 3 Lawrence Berkeley National Laboratory, Berkeley, CA, 94720, USA; 4 School of Library and Information Science, Florida State University, Tallahassee, FL, USA; 5 TUM, Department of Informatics, Bioinformatics & Computational Biology, 5748 Garching/ Munich, Germany; 6 Department of Genetics and Cambridge Systems Biology Centre, Cambridge University, Cambridge, CB2 3EH, UK; 7 eLife, Cambridge, CB2 1JP, UK; 8 Faculty of Mathematics, Computing and Technology, Open University, UK, Milton Keynes, MK7 6AA, UK; 9 Computational Biology Group, University of Cape Town, Cape Town, South Africa; 10 The Wellcome Trust Sanger Institute, Hinxton, Cambridge, CB10 1SD, UK; 11 Biomolecular Structure and Modelling Group Department of Biochemistry, University College London, London, UK; 12 Eagle Genomics Ltd, Cambridge, CB22 3AT, UK; 13 Max Planck Institute of Biochemistry, Am Klopferspitz 18, 82152, Germany; 14 TUM Graduate School of Information Science in Health (GSISH), 85748 Garching/Munich, Germany; 15 Biosof LLC, New York, NY, 10001, USA

## Abstract

BioJS is a community-based standard and repository of functional components to represent biological information on the web. The development of BioJS has been prompted by the growing need for bioinformatics visualisation tools to be easily shared, reused and discovered. Its modular architecture makes it easy for users to find a specific functionality without needing to know how it has been built, while components can be extended or created for implementing new functionality. The BioJS community of developers currently provides a range of functionality that is open access and freely available. A registry has been set up that categorises and provides installation instructions and testing facilities at http://www.ebi.ac.uk/tools/biojs/. The source code for all components is available for ready use at https://github.com/biojs/biojs.

## Commentary

In a recent press release ( http://www.nih.gov/news/health/dec2013/od-09.htm December 9 ^th^ 2013) on the occasion of the naming of Dr. Philip E. Bourne as the US NIH’s first Associate Director for Data Science, NIH director Francis S. Collins, said that “ *the era of ‘Big Data’ has arrived, and it is vital that the NIH play a major role in coordinating access to and analysis of many different data types that make up this revolution in biological information*”. We predict that one of Dr. Bourne’s main priorities will be dissemination and visualisation of biological data through the web. Web pages are ideal tools for the dissemination of results and data. Dynamic interactivity is crucial in the discovery process, particularly for data-rich applications, as is the case of many websites that provide interfaces to biological databases. Databases storing genomic and other types of data have proliferated in the biological sciences, making them a data-rich, data-intensive set of disciplines. The visualisation of these data plays a crucial role in their interpretation as it permits the ability to hide or to focus on a particular detail, enabling the researcher to shed light on specific hypotheses or to create new ones based on observed patterns. The sheer complexity of biological data, however, requires more complex technologies than the usual static pages when accessing them. They require dynamic visualisation tools to allow real-time interactions and the usability of Web 2.0-based technologies.

The JavaScript language as implemented in browsers is today’s language for the web and has transformed modern applications into client-side browser-based. JavaScript offers common Application Programming Interfaces (API) purposely built for retrieval of remote data via RESTful services, making real-time interactivity possible. JavaScript has thus drastically changed the developer/application environment, gaining competitive advantage compared to other languages. To date, there have been successful initiatives for other languages to unify open source efforts, including BioPerl ( Stajich *et al.*, 2002), BioJava ( Prlic *et al.*, 2012) or BioRuby ( Goto *et al.*, 2010). Non-language specific communities like the GMOD (Generic Model Organism Database; http://www.gmod.org/) have also enjoyed wide adoption. These initiatives provide a centralised location in which to discover available functionality, facilitating the task of finding the desired functionality.

To cater to the burgeoning JavaScript community of developers for life sciences, we created BioJavaScript (Gómez *et al.*, 2013). BioJavaScript, or BioJS for short, is a framework designed for the development and sharing of biological component visualisation on the web using JavaScript. BioJS provides a catalogue or registry to enable the user or developer to find existing functionality for reuse. Components in the registry show their current maintenance status, the contact name of the main author and a guide showing how to install, customise and extend the component. BioJS, however, is not just a repository with a set of libraries; it is also a standard set of minimum guidelines for developers to reutilise and create functionality in JavaScript as applied to biological concepts. BioJS thus provides a way for developers to build, extend and share functionality. Functionality in BioJS can be thought of as LEGO ^®^ pieces that can be connected to construct more complex applications.

## The BioJS project

**Figure 1.**
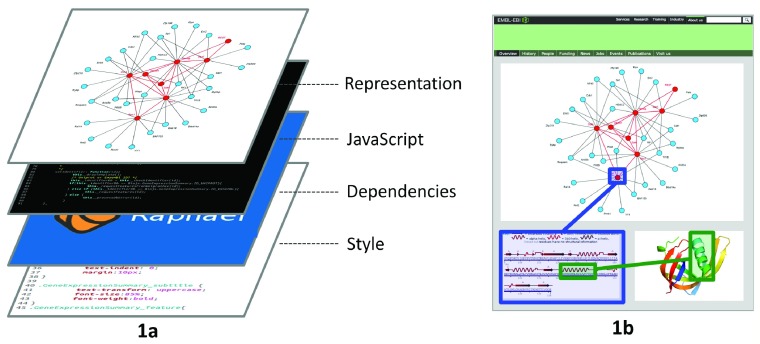
**1a** shows the different layers that a BioJS component is divided into. The representation layer sits on top of the JavaScript layer, which similarly possesses a layer of dependencies and a style. **1b** presents an example of interactivity between three components, a protein-protein interaction network viewer, a secondary structure viewer and a tertiary structure viewer. Proteins in the network are represented as nodes and their interactions as edges. Clicking on a node makes the secondary and tertiary structure viewers retrieve the same protein. It is possible to select a secondary structure element in the 2D viewer and see where it is located in the 3D visualisation component.

BioJS allows developers and users alike to i) discover functionality through its registry, currently hosted at the European Bioinformatics Institute, ii) test in the registry itself the functionality of available components, iii) reuse components in different projects and applications, iv) combine components through a common set of predefined interactions and APIs, v) customise options for each component, vi) extend the functionality of available components in a standard manner - once the developer learns how to extend a component, extension of other components is consistent, vi) maintain components via community support and documentation and vii) develop new functionality following a predefined structured architecture common to all components. The typical component contains several layers of abstraction, including a style sheet, occasionally some dependencies to libraries such as D3.js, jQuery or Raphäel, the JavaScript layer and the representation layer ( Figure 1a). Via common JavaScript event functions, several components can be integrated in a single web page, allowing for interactivity among them. For example, a three-component frame ( Figure 1b) can have a network visualisation component, with nodes representing proteins and edges representing interactions. Similarly, an alpha helix may be highlighted in a second component on the webpage that may cause a third component to highlight where the alpha helix is located in the tertiary structure of the same protein.

## The BioJS registry

At the time of writing (12-02-2014), the BioJS registry contains 39 components. The registry ( http://www.ebi.ac.uk/tools/biojs/) constitutes one of the main BioJS access portals. It contains links to documentation, the community, tutorials and the list of components. A ‘components’ page in the registry contains a current list of components available. This list includes details of the component functionality, author and current version. By clicking on one of the component links, a new page appears with a series of tabs and interactive widgets to allow the user or developer to get a feel for how the component works and looks and to find details of its installation procedure, customisation and the methods that are part of the component’s architecture, dependencies and events. Event functions can be tested on the page by using the text-box field provided. This is ideal to get a quick look and feel for the component, and it is one of the most appreciated features of the BioJS project as it makes it possible for users to test a component without needing to install it.

**Figure 2.**
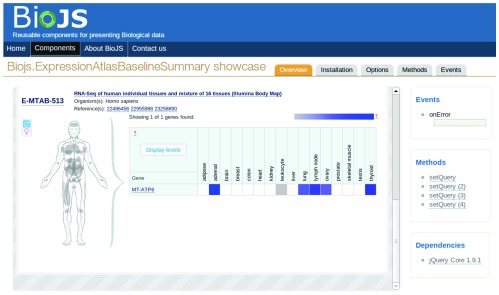
Screenshot of the *ExpressionAtlasBaselineSummary* component page in the BioJS registry, the Expression Atlas component for displaying baseline expression of genes based on RNA-seq experiments in the Expression Atlas database. Shown here is the transcription profiling by high throughput sequencing of RNA from individual and a mixture of 16 types of human tissues (E-MTAB-513 – Illumina Body Map). By default the ‘Overview’ tab is selected when accessing a registry component. On the right hand panel the prospective developer can test the main event functions (e.g., onError) and methods (e.g., setQuery). Dependencies are also shown. The installation tab provides the snippets of code required to install the component. Just copying and pasting this code should be sufficient for the component to work. The remaining tabs provide a more detailed explanation on the different options, methods and events specified for this component’s usage.

Many of the components available in the registry have been developed for particular databases. The *ExpressionAtlasBaselineSummary* component is an example of this ( Figure 2). This component was developed for the Expression Atlas database ( http://www.ebi.ac.uk/gxa/home) and subsequently deposited in the BioJS registry for reutilisation. One of the great attractive features of BioJS component visualisation is that, by sharing the component through the registry, the Expression Atlas database will ensure that other users who utilise this component will be able to enjoy a visualisation of the data exactly as intended. Having a component that can be reutilised by remote websites allows the expression data contained in this database to always be shown in the same way. This consistency in the visualisation of the same biological concepts in the same manner by different resources facilitates discovery and ease of learning by end users.

## The BioJS community

There are different degrees of involvement that users may have in BioJS. Most will utilise the web components and the registry. For those who are involved in developing biological JavaScript applications, we expect that many may find the BioJS community an attractive place to meet like-minded developers and the right environment to share their work and seek feedback and/or support. There are several mail lists currently available that reflect different levels of involvement, such as those for developers and for the Steering Committee. We also have a Twitter account (@BiojsLibrary) that informs users of news and developments. We encourage anyone interested to become involved in the way that best suits them. A number of tutorials and workshops have been organised at the European Bioinformatics Institute and elsewhere, such as the VizBi conference. We plan to organise tutorials wherever they are of service and welcome BioJS developers to become tutors whenever they wish. We have a monthly call where the Steering Committee meets, and task forces are established to reflect the needs of developers and users. Examples of task forces that have been organised include those to discuss licensing issues, compatibilities between different dependencies, and funding and usability issues. We have strong ties with the Software Sustainability Institute (SSI), a UK-based organisation that aims to provide open source community support and sustainability. SSI has recently awarded the BioJS project several months of free consultancy to make it a more robust, well-maintained and useful resource.

## Discussion

The main motivation behind BioJS is to facilitate the creation, reutilisation and sharing of JavaScript functionality across the biological domain. BioJS provides a set of minimal common guidelines and a code architecture that makes creation of new functionality more efficient and consistent. The modular structure of components makes it possible for a web framework to isolate the visualisation aspect, and thereby facilitate maintainability. The documentation that each component is required to have is based on an automatic API generator, which transforms embedded code comments (required) into a structured document. The common architecture of components makes it possible to extend them in a consistent manner: once the developer learns how to create or extend a component, the generation or extension of new ones should be straightforward. BioJS allows developers to share the development of components by making use of the community’s support. Components, once they are developed, can also be shared through the registry, so their exposure is likely to be increased. BioJS ensures that identical biological entities are visualised in the same manner and avoids different resources displaying the same content slightly differently. This reduces end-user confusion and ensures consistency across different biological domains. Having a common way in which biological entities are shown makes it easy for users to interpret the data in a more intuitive manner. BioJS can be particularly attractive to institutions that might want to have a common “flavour” for how they visualise a particular type of data. BioJS also encourages developers to aim at developing common implementations by following common guidelines on how to implement code, and makes people aware of what components are being developed by an organisation. This may constitute a competitive advantage for showcasing development contributions carried out by a particular research group or institution.

## Future directions

BioJS is a thriving community that so far has been able to attract plenty of voluntary contributions in the same spirit as other biologically-inspired open-source communities. We aim to start a series of Google Summer of Code projects to attract students interested in developing their JavaScript skills while working on life-sciences research projects. A collaborative research project has been established with the bioinformatics consultancy Eagle Genomics to develop functionality that support visualisation of genome data without a reference assembly. Many important projects and institutions have already shown commitment to the project by developing components (i.e. SwissProt ( UniProt Consortium, 2014), Genome3D ( Lewis *et al.*, 2013), Reactome ( Croft *et al.*, 2011), Expression Atlas ( Petryszak *et al.*, 2014), TGAC Browser ( http://tgac-browser.tgac.ac.uk/), etc.), and the time looks ripe to take the project to a new phase. Many challenges remain, however. The BioJS project is planning to establish a sustainable future with both robust institutional and community financial support. As the number of users increases, the need for support increases, both in terms of support to help contributors deliver but also to keep track of the state of maintenance of deposited components. Currently most of the work is done by volunteers who help fix bugs and improve the quality of submitted work. BioJS has worked well as a prototype for many projects where simple components have been created. Our first-stage mission of developing a common framework has thus been achieved. Whether we are able to meet the expectations raised by the potential of the project will only be guaranteed by the explicit commitment of important players in the bioinformatics arena.
