# Supplementary material for: BioJS: an open source standard for biological visualisation – its status in 2014
Source: F1000Res. 2014 Feb 13;3:55. [Version 1] doi: 10.12688/f1000research.3-55.v1 (PMC4103492; doi:10.12688/f1000research.3-55.v1)
Supplement: Peer review history

# Peer review: Referee response for version 1

**DOI:** 10.5256/f1000research.3850.r3706

**Published:** 2014-03-17

**Authors:** Reinhard Schneider

**Affiliations:** 1 University of Luxemburg, Luxembourg, USA

Visualization is one of the bottlenecks we are facing in modern biology. With bigger and bigger data sets originating from various heterogeneous sources that span several concepts, dimensions and timelines, it is becoming an art to condense the wealth of information into good and easy ways to capture visual pictures.

For non-expert users it can be quite a challenge to come up with a "publication ready" visualization of complex relationships. The BioJS community tries to tackle this challenge by employing an open modular architecture, which allows a broad audience to use and further develop the components in the registry. The community already provides a wide spectrum of available components and it will be very interesting to see the future development of this open source community.

I have read this submission. I believe that I have an appropriate level of expertise to confirm that it is of an acceptable scientific standard.

# Peer review: Referee response for version 1

**DOI:** 10.5256/f1000research.3850.r3819

**Published:** 2014-02-20

**Authors:** Phil Bourne

**Affiliations:** 1 National Institutes of Health (NIH), Bethesda, ML, USA

I am supportive of the BioJS registry with regards to the technology used and its principles. To give you a flavour of what I will be looking for, from an NIH perspective, is really how much the components are used and why. What findings have resulted in their use? If there is non-use of components and why etc. In short, I will be looking more at the degree of software projects that are making a difference to the scientific community. That said, I very much look forward to seeing how the BioJS community develops.

I have read this submission. I believe that I have an appropriate level of expertise to confirm that it is of an acceptable scientific standard.
